# DNA methylation variations induced by salt and alkali stresses and their differential responses in *Tamarix chinensis* L

**DOI:** 10.3389/fpls.2026.1894569

**Published:** 2026-08-05

**Authors:** Long Wang, Bo Li, Yue Wang, Yuqian Wang, Jianpeng Wang, Mengke Yang, Haoxuan Li, Hongyan Wang

**Affiliations:** 1 Laboratory of Plant Epigenetics and Evolution, School of Life Sciences, Liaoning University, Shenyang, China; 2 Academy of Agricultural and Forestry Sciences, Qinghai University, Xining, China

**Keywords:** salt stress, alkali stress, DNA methylation, methylation‑sensitive amplified polymorphism (MSAP), *Tamarix chinensis* L.

## Abstract

Soil salinization is a critical environmental challenge threatening plant survival. To adapt, certain plants have evolved tolerance to highly saline-alkali environments through long-term natural selection. *Tamarix chinensis* L., a typical halophyte with strong adaptability to salt and alkali stresses, serves as an essential germplasm resource and pioneer species for ecological conservation and remediation. However, the epigenetic mechanisms underlying its stress tolerance remain poorly understood. In this study, we systematically characterized the tolerance and genome-wide DNA methylation variations in *T. chinensis* under diverse salt and alkali stresses conditions. Phenotypic observations manifested severe leaf damage under 150 mM alkali stress over 10 days, whereas only slight injury occurred under 400 mM salt stress, indicating superior tolerance to salinity over alkalinity. MSAP analysis revealed that salt stress predominantly triggered genome-wide demethylation; conversely, alkali stress primarily induced hypermethylation, with both variations occurring mainly at CNG sites. Notably, DNA methylation alterations were more extensive under salt stress. Sequencing of methylation-variant bands yielded 17 stress-related sequences, eight of which are homologous to known stress-responsive genes. These findings provide a theoretical framework for dissecting differential epigenetic responses to salt and alkali stresses, facilitate the mining of resistance genes, and offer epigenetic references for the molecular breeding of *T. chinensis* to and ecological remediation of saline-alkaline lands.

## 1 Introduction

Soil salinization represents a major constraint on global agricultural productivity and ecological conservation, serving as a predominant abiotic stressor that restricts the efficient utilization of arable land, particularly in saline-alkali regions (Deeb et al., 2024; Chen et al., 2025). Globally, salinized land covers approximately one billion hectares, accounting for 7% of the total landmass (Bian et al., 2021). Secondary salinization affects an additional 77 million hectares, with roughly 58% occurring in irrigated farmland (Kasim et al., 2020; Sun et al., 2020). Excessive salinization exacerbates soil compaction and severely inhibits plant growth and development, potentially leading to widespread mortality and substantial economic losses in sustainable agriculture (Rumsey et al., 2021). Salt stress damage primarily stems from excessive accumulation of Na^+^ and Cl^−^, which lowers soil water potential and hinders plant water absorption (Hasegawa et al., 2000). Alkali stress, however, inflicts even more severe damage. Its high pH environment triggers precipitation of soil ions, including Ca^2+^, Mg^2+^, and HPO_3_^−^, thereby disrupting plant ion balance and tissue homeostasis, which subsequently impairs inorganic ion absorption (Martinez-Alonso et al., 2024). Consequently, enhancing the salt and alkali tolerance of plants is of profound significance for both agricultural development and ecological remediation.

Dynamic environmental changes severely affect plant growth and development; consequently, plants rely on phenotypic plasticity and genetic diversity to adapt to such stressors (Williams et al., 2023; Li et al., 2025). As a well-characterized epigenetic modification (Lu et al., 2025; Pei et al., 2025), DNA methylation regulates plant growth, development, genomic imprinting, and genome stability, while playing crucial roles in stress responses (Slatkin, 2009; Feng and Jacobsen, 2011; Garg et al., 2015). This modification involves the transfer of a methyl group from S-adenosylmethionine to the 5-position of cytosine by methyltransferases, forming 5-methylcytosine (Santi et al., 1983). It occurs across three distinct sequence contexts: CG, CHG, and CHH (where H represents A, T, or C) (Deaton and Bird, 2011). Dynamic shifts in DNA methylation modulate the transcription of stress-responsive genes, thereby facilitating adaptation to abiotic challenges (Zhang et al., 2018; Song et al., 2025; Wu et al., 2025). Numerous studies have corroborated the critical role of DNA methylation in plant stress resilience. For instance, using methylation-sensitive amplification polymorphism (MSAP) analysis, researchers found that waterlogging stress induced extensive demethylation in wheat, identifying three demethylated genes (*ERF1*, *ACC1*, and *CKX2.3*) whose expression patterns closely correlated with stress tolerance (Pan et al., 2020). Similarly, a comparative analysis of salt-tolerant (FL478) and salt-sensitive (IR29) rice varieties revealed that salt stress decreased root DNA methylation levels in both genotypes, with a more pronounced reduction observed in the sensitive variety. Sequencing further revealed that these methylation -variant fragments shared high homology with stress-related proteins, including Ser/Thr phosphatases, NB-ARC domain-containing proteins, OsWRKY transcription factors, and zinc finger domain proteins (Wang et al., 2011). Furthermore, pre-treating kenaf (*Hibiscus cannabinus* L.) seedlings with 5-azacytidine (5-azaC) prior to salt stress significantly elevated antioxidant enzyme activities and mitigated ROS accumulation, while MSAP results showed reduced DNA methylation levels, suggesting kenaf seedlings enhance stress resilience via demethylation modifications (Li et al., 2021). Additionally, researchers demonstrated that high-concentration ozone stress induces extensive whole-genome demethylation variations in rice, thereby mediating adaptive responses (Wang et al., 2023). Collectively, these studies indicate DNA methylation serves as a rapid and dynamic epigenetic mechanism for plants confronting abiotic stress, playing a vital role in environmental adaptation.

*T*. *chinensis*, a woody halophyte within the Tamaricaceae family, possesses exceptional tolerance to saline-alkaline, drought, and high-temperature stressses (Qu et al., 2024; Yang et al., 2024). Its unique salt gland structures facilitate adaptation to diverse hypersaline environments (Ding et al., 2010). Previous studies have indicated that key transcription factors, including bHLH, WRKY, MYC, and AP2/ERF, play key roles in stress responses in *T*. *chinensis*, contributing to salt tolerance by regulating phytohormone signal transduction pathways (Zheng et al., 2013; Wang et al., 2014; Ji et al., 2016). Through the analysis of expressed sequence tags (ESTs) for *T*. *chinensis*, researchers have identified numerous salt- and alkali-stress-responsive genes, confirming that this robust tolerance trait emerges from the synergistic interactions among multiple genetic components and physiological-biochemical pathways (Gao et al., 2008). However, while existing research has elucidated certain tolerance mechanisms concerning physiological indices and gene expression, the underlying epigenetic regulations remain largely unexplored, particularly regarding the dynamic and differential DNA methylation patterns under salt versus alkali stress.

Consequently, employing MSAP technology, this study systematically investigated the levels and patterns of genomic DNA methylation variation in *T*. *chinensis* subjected to salt and alkali stresses. We aim to comprehensively characterize the epigenetic divergence in its responses to these two distinct stressors. This research intends to provide a novel theoretical framework for understanding the epigenetic basis of stress tolerance in *T*. *chinensis*, offering valuable insights into the evolutionary adaptability and unique tolerance mechanisms of saline-alkali-tolerant plants.

## 2 Materials and methods

### 2.1 Plant material and salt-alkali stress treatments

One-year-old *T*. *chinensis* branches were cut into 15 cm segments with 1 cm diameters and planted in pots containing a 1:2:1 (v:v:v) mixture of peat, vermiculite, and perlite. Cultivation was conducted in a walk-in growth chamber maintained at 25 °C with 65%–70% relative humidity under a 15 h light/9 h dark photoperiod. After 50 days, uniform seedlings were selected for subsequent stress experiments. Salt stress was induced using a 1:1 molar mixture of Na_2_SO_4_ and NaCl) at concentrations of 100, 200, 300, and 400 mmol·L^−^¹. Similarly, alkali stress treatments employed a 1:1 molar mixture of Na_2_CO_3_ and NaHCO_3_ at concentrations of 50, 100, 150, and 200 mmol·L^−^¹. These salt and alkali agents were integrated into half-strength Hoagland nutrient solutions to form the final treatment media, while an unmodified half-strength Hoagland solution served as the control. Each treatment group consisted of three biological replicates, with each pot containing four individual plants. Leaf tissues were harvested at 5, 10, 20, and 30 days post-treatment for downstream DNA methylation analysis.

### 2.2 Genomic DNA methylation analysis of *T*. *chinensis* Leaves under salt and alkali stresses

Genomic DNA was isolated from *T*. *chinensis* leaves using a modified CTAB protocol (Kidwell and Osborn, 1992). MSAP analysis was then employed to evaluate DNA methylation levels, variation patterns, and modification sites (Wang et al., 2023). The specific sequences for primers and adapters utilized in this study are listed in **Supplementary Table 1**. Cytosine methylation at CCGG sites was categorized into four distinct patterns: Type I (unmethylated), Type II (hemi-methylated), and Types III and IV (both representing fully methylated states). Band presence and absence were scored as ‘1’ and ‘0’, respectively, and banding patterns were analyzed separately for the *EcoRI* + *HpaII* and *EcoRI* + *MspI* digestion combinations. The total methylation level (MSAP%) was quantified according to the following formula:


MSAP(%)=[(II+ III +IV)/(I+II+III+IV)]×100%

### 2.3 MSAP procedure and scoring of methylation bands

MSAP analysis was performed according to established protocols with modifications. Genomic DNA (500 ng) was digested with EcoRI (20 U) in combination with either HpaII (10 U) or MspI (10 U) at 37 °C for 6 h, followed by enzyme inactivation at 65 °C for 20 min. Complete digestion was verified by agarose gel electrophoresis, which showed a uniform smear pattern, using undigested DNA as control. The digested products were ligated to EcoRI and HpaII/MspI adapters using T4 DNA ligase (40 U) at 8 °C for 4 h and then stored at 4 °C overnight. Pre-amplification was carried out in a reaction mixture containing DNA template (2 μL), 10× PCR buffer (2 μL), dNTPs (1 μL), EcoRI+A and HpaII/MspI+0 primers (5 μM, 1 μL each), rTaq polymerase (0.2 μL), and ddH_2_O (12.8 μL), for 30 cycles at an annealing temperature of 56 °C. The pre-amplification products were verified by agarose gel electrophoresis, which showed bright and smeared bands at approximately 500 bp. Subsequently, selective amplification was performed using primers with two selective nucleotides (E-AAC, E-AAG, E-ACA, E-ACT, E-ACC, E-ACG, E-AGC, E-AGG, E-AGA, E-ATC; HM-TCT, HM-TCG, HM-TCC, HM-TTC, HM-TTG, HM-TTA, HM-TGA, HM-TGT, HM-TGC, HM-TAC; 5 μM, 0.6 μL each), diluted pre-amplification template (1.2 μL), 10× PCR buffer (1.5 μL), dNTPs (0.6 μL), rTaq polymerase (0.12 μL), and ddH_2_O (10.38 μL), using a touchdown PCR program (65 °C decreasing to 55 °C) for 35 cycles. The selective amplification products were separated on 5% denaturing polyacrylamide gels, fixed with glacial acetic acid, silver-stained with silver nitrate, and developed with sodium bicarbonate and formaldehyde. All experiments were performed with three independent biological replicates, and only stable and clear bands were recorded for analysis.

### 2.4 Ligation, transformation, and sequencing of the DNA methylation variation band

Differential DNA methylation bands were excised from the polyacrylamide gels and equilibrated in TE buffer at 37 °C for 8 hours. Following centrifugation, the resulting supernatants were collected to serve as templates for PCR amplification. The purified amplification products were then ligated into pUM19-T vectors (Shanghai Sangon Biotech Co., Ltd., Shanghai, China) and subsequently transformed into DH-5α competent cells (Beijing Dingguo Changsheng Biotechnology Co., Ltd., Beijing, China). Finally, single clones were selected for Sanger sequencing.

### 2.5 DNA methylation variation sequence homology analysis

BioEdit (v.7.0.5) was employed to screen primers and trim vector sequences from the obtained methylation-variant fragments. The filtered sequences were subsequently subjected to homology searches via BlastN and BlastX algorithms. and functional analyses were performed using the EnsemblPlants (http://plants.ensembl.org) and NCBI (http://blast.ncbi.nlm.nih.gov) databases.

## 3 Results

### 3.1 Visible damage induced by salt and alkali stresses in *T*. *chinensis*

Abiotic stress-induced damage is most visibly manifested in plant leaves, where changes in color and morphology serve as reliable indicators of stress severity (Hura et al., 2022). Following exposure to varying concentrations of salt and alkali, the leaf damage characteristics of *T. chinensis* were systematically monitored and recorded across all treatments.

**Figure 1:**
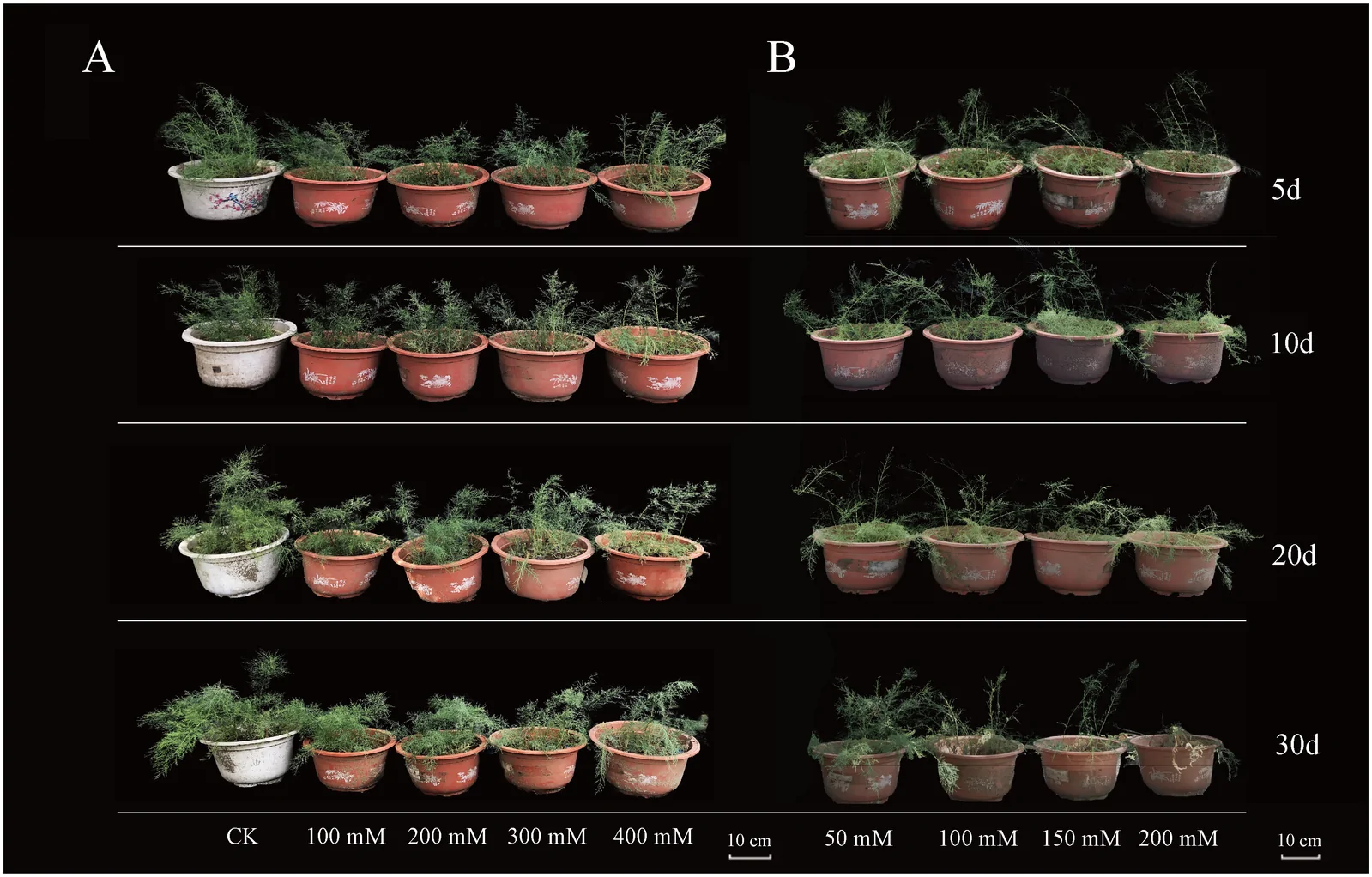
Growth status of *T*. *chinensis* under salt and alkali stresses. **(A)** Seedlings subjected to salt stress across varying concentrations (100, 200, 300, and 400 mM) and durations (5, 10, 20, and 30 days). **(B)** Seedlings subjected to alkali stress across varying concentrations (50, 100, 150, and 200 mM) and durations (5, 10, 20, and 30 days).

Under salt stress, the phenotypic damage in *T*. *chinensis* exhibited time- and concentration-dependent characteristics. During the initial 5 days, plants across all salt levels remained largely unaffected, maintaining vibrant green leaves without detectable chlorosis or yellowing. By day 10, only the 400 mM group displayed slight chlorosis, while other groups remained visibly healthy. After 20 days, although the 100–300 mM groups maintained normal phenotypes, the 400 mM group showed intensified leaf chlorosis, with foliage turning pale. By day 30, damage in the 400 mM group progressed to severe chlorosis, with leaves appearing dark yellow (**Figure 1A**). These observations suggest that *T*. *chinensis* possesses robust salt tolerance, capable of sustaining prolonged growth under low-to-moderate salinity.

In contrast, alkali stress induced more pronounced and accelerated damage in *T*. *chinensis*, albeit still following a time- and dose-dependent trajectory (**Figure 1B**). By day 10, leaves in the 150 mM and 200 mM alkali groups already exhibited evident chlorosis. This damage exacerbated by day 20 as chlorotic areas expanded, while the 100 mM group also began to develop symptoms. By day 30, higher alkali concentrations had progressively worsened leaf condition, ultimately culminating in widespread yellowing and desiccation. Overall, these results demonstrate that *T*. *chinensis* exhibits significantly higher tolerance to salt than to alkali, as the latter triggers swifter and more severe phenotypic damage.

### 3.2 Extensive genomic DNA methylation variations in *T*. *chinensis* under salt and alkali stresses

**Figure 2:**
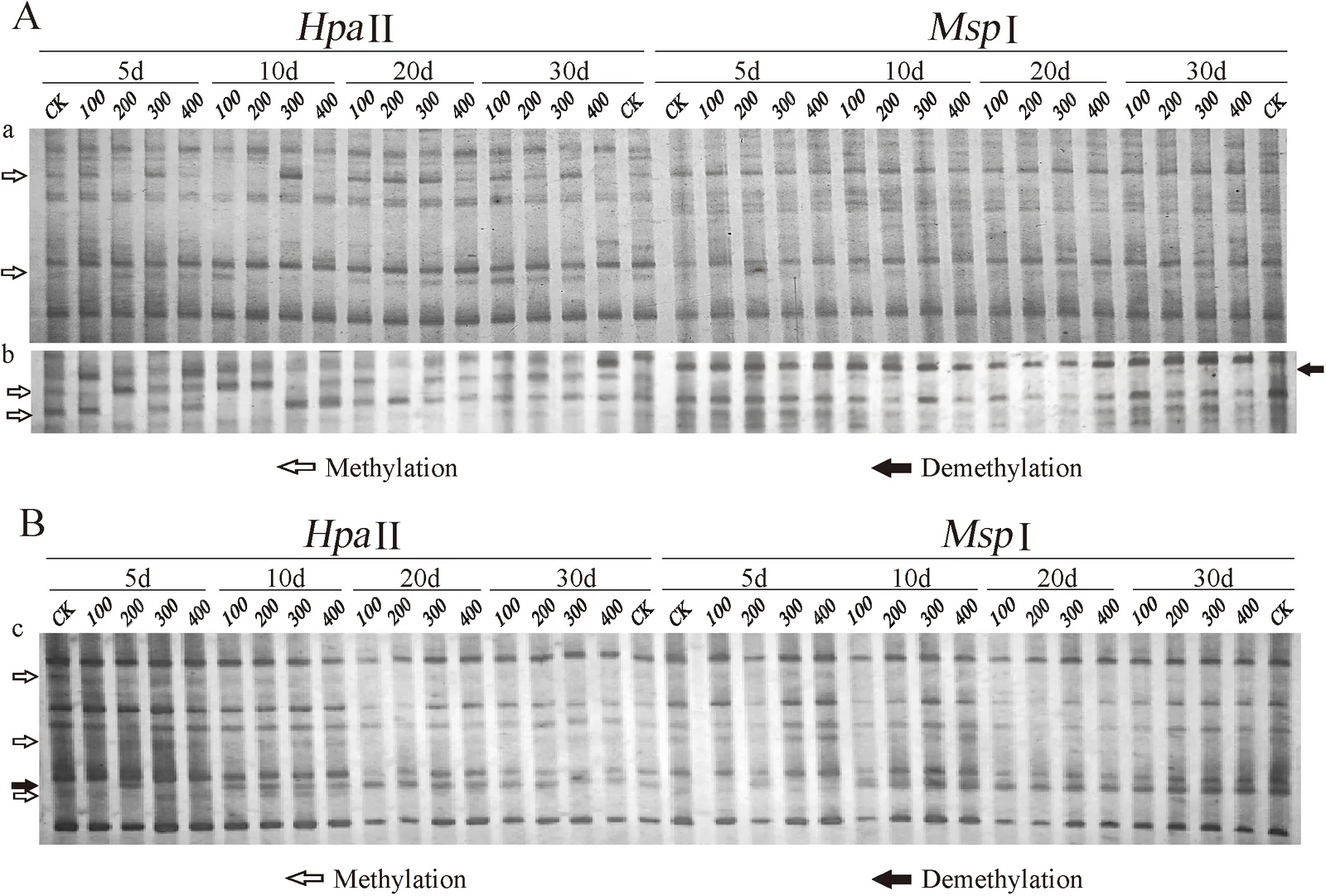
DNA methylation variation patterns in *T*. *chinensis* under salt and alkali stresses. **(A)** Seedlings subjected to salt stress across varying concentrations (CK, 100, 200, 300, and 400 mM) and durations (5, 10, 20, and 30 days). **(B)** Seedlings subjected to alkali stress across varying concentrations (CK, 50, 100, 150, and 200 mM) and durations (5, 10, 20, and 30 days). Primer combinations: *Eco*RI + ATC/*Hpa*II(*Msp*I) + TTC (a), *Eco*RI + AAC/*Hpa*II(*Msp*I) + TGC (b), *Eco*RI + ACG/*Hpa*II(*Msp*I) + TCG (c).

To elucidate the epigenetic regulatory mechanisms in *T*. *chinensis* under salt and alkali stresses, MSAP analysis was employed to quantify genome-wide DNA methylation across various stress intensities and durations. Under salt stress, MSAP profiling yielded between 1103 and 1134 amplification bands, identifying a total of 1167 clearly distinguishable methylation sites (**Table 1**; **Figure 2A**). Based on restriction enzyme combination differences, these sites were categorized into four groups: unmethylated (Type I, 933–976 bands, 79.95%–83.63%), hemi-methylated (Type II, 51–83 bands, 4.37%–7.11%), fully methylated (Types III and IV, 127–159 bands, 10.88%–13.62%), and total methylated (Types II+III+IV, 191–234 bands, 16.37%–20.05%). Compared to the control group (20.39%), total methylation decreased across all salt treatments, indicating that salt stress predominantly triggers genomic demethylation in *T*. *chinensis*.

**Table 1:** Cytosine methylation levels in *T. chinensis* under varying salt stress treatments as determined by MSAP analysis.

Type	Salt Stress Time-Point																	
	5- CK	5-100	5-200	5-300	5-400	10- 100	10- 200	10-300	10-400	20-100	20-200	20-300	20-400	30-100	30-200	30- 300	30- 400	30- CK
I	929	969	962	955	944	941	933	939	940	934	933	941	948	952	956	959	976	929
II	79	71	75	76	80	78	75	75	76	78	83	78	69	64	62	52	51	79
III	95	94	96	94	105	105	116	107	105	103	111	109	114	109	105	112	99	95
IV	64	33	34	42	38	43	43	46	46	52	40	39	36	42	44	44	41	64
Total sites	1167	1167	1167	1167	1167	1167	1167	1167	1167	1167	1167	1167	1167	1167	1167	1167	1167	1167
Total amplified bands	1103	1134	1133	1125	1129	1124	1124	1121	1121	1115	1127	1128	1131	1125	1123	1123	1126	1126
Total methylated bands	238	198	205	212	223	226	234	228	227	233	234	226	219	215	211	208	191	238
MSAP (%)	20.39	16.97	17.57	18.17	19.11	19.37	20.05	19.54	19.45	19.97	20.05	19.37	18.77	18.42	18.08	17.82	16.37	20.39
Fully methylated bands	159	127	130	136	143	148	159	153	151	155	151	148	150	151	149	156	140	159
Fully methylated ratio %	13.62	10.88	11.14	11.65	12.25	12..68	13.62	13.11	12.94	13.28	12.94	12.68	12.85	12.94	12.77	13.37	12	13.62
Hemi-methylated ratio %	6.77	6.08	6.43	6.51	6.86	6.68	6.43	6.43	6.51	6.68	7.11	6.68	5.91	5.48	5.31	4.46	4..37	4.46
Non-methylated ratio%	79.61	83.03	82.43	81.83	80.89	80.63	79.95	80.46	80.55	80.03	79.95	80.63	81.23	81.58	81.92	82.18	83.63	79.61

^a^ MSAP(%)=[(II+III+IV)/(I+II+III+IV)] × 100.

^b^ Fully methylated ratio(%)=[(III+IV)/(I+II+III+IV)] × 100.

^c^ Hemi-methylated ratio(%)=[(II)/(I+II+III+IV)] × 100.

^d^ Non-methylated ratio(%)=[(I)/(I+II+III+IV)] × 100.

In contrast, alkali stress yielded 1178–1193 bands, corresponding to 1228 distinct methylation sites (**Table 2**; **Figure 2B**). The site distribution was as follows: unmethylated (Type I, 934–975 bands, 76.06%–79.40%), hemi-methylated (Type II, 73–111 bands, 5.94%–9.04%), fully methylated (Types III and IV, 175–199 bands, 14.25%–16.21%), and total methylated (Types II+III+IV, 253–294 bands, 20.60%–23.94%). The genomic methylation variations in *T*. *chinensis* under alkali stress exhibited a more complex trajectory than the linear demethylation observed under salt stress. Initially, methylation levels rose from 21.17% in controls to a peak of 23.94% (5 d, 50 mM). Although levels gradually declined to 20.60% by day 30 (50 mM), the overall methylation remained generally higher than that of the controls. These results suggest that alkali stress primarily induces a genomic hypermethylation response in *T*. *chinensis*.

**Table 2:** Cytosine methylation levels in *T. chinensis* under varying alkali stress treatments as determined by MSAP analysis.

Type	Alkali Stress Time-Point																	
	5- CK	5- 50	5- 100	5- 150	5- 200	10- 50	10- 100	10- 150	10- 200	20- 50	20- 100	20- 150	20- 200	30- 50	30- 100	30- 150	30- 200	30- CK
I	968	934	943	944	942	954	945	946	944	945	958	964	970	975	973	962	956	968
II	78	109	101	100	111	94	87	85	97	96	84	89	82	75	75	78	73	78
III	142	143	142	136	134	136	146	148	146	148	143	140	138	136	139	148	156	142
IV	40	42	42	48	41	44	50	49	41	39	43	35	38	42	41	40	43	40
Total sites	1228	1228	1228	1228	1228	1228	1228	1228	1228	1228	1228	1228	1228	1228	1228	1228	1228	1228
Total amplified bands	1188	1186	1186	1180	1187	1184	1178	1179	1187	1189	1185	1193	1190	1186	1187	1188	1185	1188
Total methylated bands	260	294	285	284	286	274	283	282	284	283	270	264	258	253	255	266	272	260
MSAP (%)	21.17	23.94	23.21	23.13	23.29	22.31	23.05	22.96	23.13	23.05	21.99	21.50	21.01	20.60	20.77	21.66	22.15	21.17
Fully methylated bands	182	185	184	184	175	180	196	197	187	187	186	175	176	178	180	188	199	182
Fully methylated ratio %	14.82	15.06	14.98	14.98	14.25	14.66	15.96	16.04	15.23	15.23	15.15	14.25	14.33	14.50	14.66	15.31	16.21	14.82
Hemi-methylated ratio %	6.35	8.88	8.22	8.14	9.04	7.65	7.08	6.92	7.90	7.82	6.84	7.25	6.68	6.11	6.11	6.35	5.94	6.35
Non-methylated ratio%	78.83	76.06	76.79	76.87	76.71	77.69	76.95	77.04	76.87	76.95	78.01	78.5	78.99	79.4	79.23	78.34	77.85	78.83

^a^ MSAP(%)=[(II+III+IV)/(I+II+III+IV)] × 100.

^b^ Fully methylated ratio(%)=[(III+IV)/(I+II+III+IV)] × 100.

^c^ Hemi-methylated ratio(%)=[(II)/(I+II+III+IV)] × 100.

^d^ Non-methylated ratio(%)=[(I)/(I+II+III+IV)] × 100.

In summary, salt and alkali stresses elicit divergent epigenetic responses in *T*. *chinensis*. While salt stress is characterized by a predominant trend of DNA demethylation, alkali stress tends to promote DNA hypermethylation.

### 3.3 DNA methylation variation patterns in *T*. *chinensis* under salt and alkali stresses

**Figure 3:**
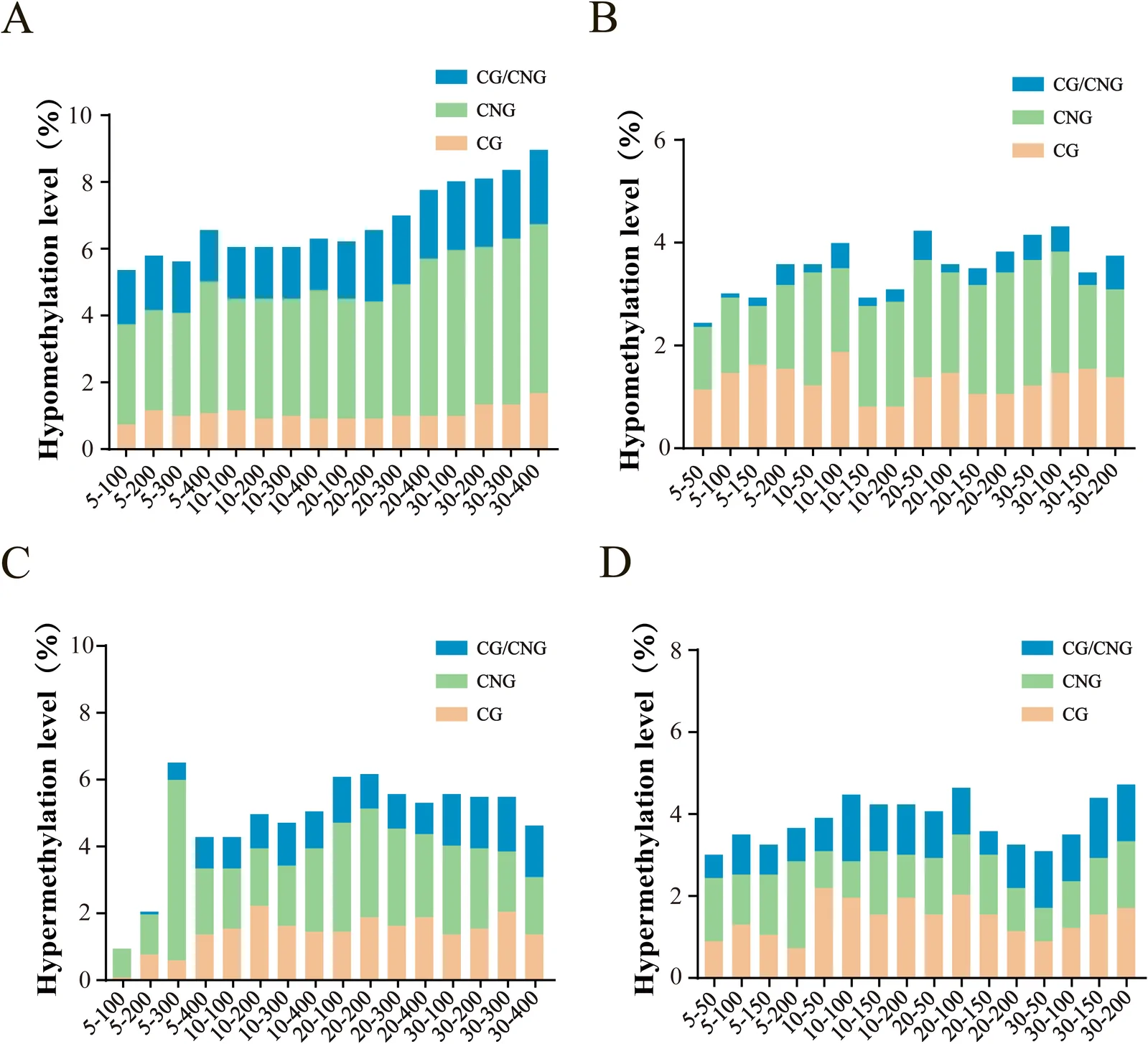
Statistics of DNA methylation -variant sites in *T*. *chinensis* under salt and alkali stresses. **(A)** Demethylation -variant sites under salt stress. **(B)** Demethylation -variant sites under alkali stress. **(C)** Hypermethylation-variant sites under salt stress. **(D)** Hypermethylation -variant sites under alkali stress.

To further elucidate the patterns and dynamics of genomic DNA methylation in *T*. *chinensis* under stress, the distribution and response characteristics of demethylation and hypermethylation sites were systematically evaluated. Under salt stress, demethylation frequencies increased progressively with both treatment duration and concentration, ranging from 5.40% to 9.00% (**Table 3**). For instance, the 100 mM group showed a gradual increase from 5.40% at day 5 to 8.05% at day 30, with similar upward trends observed across all salt concentrations. The highest demethylation frequency (9.00%) was observed in the 400 mM group at day 30, while the lowest (5.40%) occurred in the 100 mM group at day 5. These data manifest a clear synergistic effect between stress duration and dosage, as demethylation intensified with increasing salt concentration and treatment time. Regarding variation patterns, variant sites were distributed predominantly across CNG and CG/CNG sites, followed by CG sites, representing 1.29%–3.17%, 1.54%–2.23%, and 0.94%–2.06% of the total sites, respectively (**Figure 3A**). In contrast, the magnitude of demethylation under alkali stress was notably lower, ranging from 2.44% to 4.32% (**Table 4**). Relative to controls, demethylation frequencies in the 50, 100, 150, and 200 mM groups rose from 2.44%, 3.01%, 2.93%, and 3.58% at day 5 to 4.15%, 4.32%, 3.42%, and 3.75% by day 30, respectively. Unlike the salt -stress patterns, demethylation variant sites under alkali stress were primarily concentrated in CNG and CG sites, followed by CG/CNG sites, representing 1.14%–2.44%, 0.81%–1.87%, and 0.08%–0.65% of the total sites, respectively (**Figure 3B**).

**Table 3:** DNA methylation variation patterns in *T*. *chinensis* under salt stress.

Banding Pattern						Different Treatment															
Pattern	Class Control Salt																				
		H	M	H	M	5- 100	5- 200	5- 300	5- 400	10- 100	10- 200	10- 300	10-400	20-100	20-200	20-300	20- 400	30-100	30- 200	30-300	30-400
	A	1	1	1	1	922	915	866	892	891	885	892	888	881	877	883	885	887	888	891	902
	B	1	0	1	0	60	60	58	51	55	52	50	46	45	48	47	42	35	36	32	32
NO Change	C	0	1	0	1	82	76	74	76	75	78	77	76	73	75	74	75	75	71	72	67
	D	0	0	0	0	44	44	42	36	41	39	40	39	39	33	32	31	30	33	33	31
Total(%)						1108	1095	1040	1055	1062	1054	1059	1049	1038	1033	1036	1033	1027	1028	1028	1032
						94.94	93.83	89.12	90.40	91.00	90.32	90.75	89.89	88.95	88.52	88.77	88.52	88.00	88.09	88.09	88.43
	E	1	0	1	1	19	14	17	21	18	19	17	23	22	20	22	27	29	28	28	28
	F	0	1	1	1	9	14	12	13	14	11	12	11	11	11	12	12	12	16	16	20
Hypomethylation	G	0	0	1	1	19	19	18	18	18	18	18	18	20	25	24	24	24	24	24	26
	H	0	0	1	0	4	4	5	11	7	9	9	9	6	6	6	6	6	5	5	5
	I	0	0	0	1	11	11	13	13	12	12	11	12	13	14	16	17	18	16	16	16
	J	1	0	0	1	0	4	0	0	1	2	3	1	1	1	2	5	5	5	8	8
	K	0	1	1	0	1	2	1	1	1	0	1	0	0	0	0	0	0	1	1	2
Total (%)						63	68	66	77	71	71	71	74	73	77	82	91	94	95	98	105
						5.40	5.83	5.66	6.60	6.08	6.08	6.08	6.34	6.26	6.60	7.03	7.80	8.05	8.14	8.40	9.00
	L	1	1	1	0	6	9	54	17	15	14	15	21	27	29	25	21	23	20	14	12
	M	1	1	0	1	1	5	7	16	17	24	16	16	16	21	17	17	11	13	16	8
Hypermethylation	N	1	0	0	1	0	4	0	0	1	2	3	1	1	1	2	5	5	5	8	8
	O	1	1	0	0	0	0	2	4	6	6	6	4	5	2	4	6	8	8	8	7
	P	1	0	0	0	0	1	4	7	5	6	9	9	11	10	8	5	10	10	11	11
	Q	0	1	0	0	3	3	8	5	5	6	5	8	11	9	9	8	8	7	6	6
	R	0	1	1	0	1	2	1	1	1	0	1	0	0	0	0	0	0	1	1	2
Total (%)						11	24	76	50	50	58	55	59	71	72	65	62	65	64	64	54
						0.94	2.06	6.51	4.28	4.28	4.97	4.71	5.06	6.08	6.17	5.57	5.31	5.57	5.48	5.48	4.63

**Table 4:** DNA methylation variation patterns in *T*. *chinensis* under alkali stress.

Banding Pattern						Different Treatment															
Pattern	Class Control Salt																				
		H	M	H	M	5- 50	5- 100	5- 150	5- 200	10- 50	10- 100	10-150	10-200	20-50	20-100	20-150	20- 200	30-50	30-100	30-150	30-200
	A	1	1	1	1	902	903	905	903	907	897	899	898	897	905	911	916	914	912	907	909
	B	1	0	1	0	129	123	121	120	118	119	125	120	123	119	122	121	117	117	118	123
NO Change	C	0	1	0	1	63	68	69	71	70	67	67	68	68	68	68	68	68	67	68	68
	D	0	0	0	0	33	26	35	25	26	27	31	24	22	24	23	20	20	22	24	24
Total(%)						1127	1120	1130	1119	1121	1110	1122	1110	1110	1116	1124	1125	1119	1118	1117	1124
						91.78	91.21	92.02	91.12	91.29	90.39	91.37	90.39	90.39	90.88	91.53	91.61	91.12	91.04	90.96	91.53
	E	1	0	1	1	5	4	5	5	9	7	6	8	10	5	7	8	10	12	4	2
	F	0	1	1	1	13	17	18	16	12	21	8	8	15	16	13	13	15	18	19	17
Hypomethylation	G	0	0	1	1	1	1	2	5	2	6	2	3	7	2	4	5	6	6	3	8
	H	0	0	1	0	7	10	5	10	4	8	6	5	9	6	11	10	10	9	2	4
	I	0	0	0	1	3	3	2	4	9	3	10	8	6	8	6	9	8	7	7	11
	J	1	0	0	1	0	1	2	1	5	2	2	4	3	5	2	2	2	1	7	4
	K	0	1	1	0	1	1	2	3	3	2	2	2	2	2	0	0	0	0	0	0
Total (%)						30	37	36	44	44	49	36	38	52	44	43	47	51	53	42	46
						2.44	3.01	2.93	3.58	3.58	3.99	2.93	3.09	4.23	3.58	3.5	3.83	4.15	4.32	3.42	3.75
	L	1	1	1	0	16	10	12	17	6	6	14	8	12	14	12	8	5	6	14	12
	M	1	1	0	1	11	15	11	8	19	22	16	19	18	19	14	12	9	14	16	18
Hypermethylation	N	1	0	0	1	0	1	2	1	8	2	3	5	1	6	5	2	2	1	3	3
	O	1	1	0	0	3	4	4	4	6	7	9	11	5	8	3	5	4	3	10	9
	P	1	0	0	0	4	8	5	6	4	13	5	4	9	6	4	8	13	11	8	8
	Q	0	1	0	0	2	4	4	6	2	3	3	3	3	2	5	5	5	5	3	8
	R	0	1	1	0	1	1	2	3	3	2	2	2	2	2	1	0	0	3	0	0
Total (%)						37	43	40	45	48	55	52	52	50	57	44	40	38	43	54	58
						3.01	3.50	3.26	3.66	3.91	4.48	4.23	4.23	4.07	4.64	3.58	3.26	3.09	3.50	4.40	4.72

Hypermethylation analysis indicated that *T*. *chinensis* showed genomic hypermethylation under salt stress, with frequencies ranging from 0.94% to 6.51% **(Table 3**). Compared to controls, hypermethylation in the 100 mM group rose from 0.94% at day 5 to 5.57% by day 30, while the 200 mM group increased from 2.06% to 5.48%. Notably, the 300 mM group showed a slight contraction, decreasing from 6.51% to 5.48%, whereas the 400 mM group exhibited a modest elevation from 4.28% to 4.63%. Regarding sequence contexts, hypermethylated variant sites were predominantly localized at CNG and CG motifs, followed by CG/CNG sites, accounting for 0.94%–5.48%, 0.09%–2.74%, and 0.09%–1.63% of total variant sites, respectively (**Figure 3C**). Under alkali stress, hypermethylation frequencies fluctuated between 3.01% and 4.72% (**Table 4**). Compared with controls, over the 30-day treatment period, the 50 mM group remained relatively stable, with only a slight increase from 3.01% at day 5 to 3.09% at day 30. The 100 mM group exhibited a distinct transient increase, rising from 3.50% at day 5 to a peak of 4.64% at day 20, followed by a decline back to 3.50% at day 30. In contrast, the 150 mM and 200 mM groups showed sustained upward trends over time, increasing from 3.26% and 3.66% at day 5 to 4.40% and 4.72% by day 30, respectively. Regarding variation patterns, alkali-induced hypermethylated sites were distributed predominantly across CG and CNG contexts, followed by CG/CNG sites, representing 0.73%–2.20%, 0.81%–2.12%, and 0.57%–1.63% of the total sites, respectively (**Figure 3D**).

Collectively, these findings demonstrate that varying intensities of salt and alkali stresses trigger extensive genomic DNA remodeling in *T*. *chinensis*, generally manifesting synergistic time- and dosage-dependent effects. While salt stress was characterized by a predominance of DNA demethylation, alkali stress primarily elicited a hypermethylation response. In both scenarios, demethylation occurred mainly at CNG sites; however, hypermethylation targeted CNG sites under salt stress but favored CG sites under alkali stress. Compared to alkali stress, methylation variation magnitudes were significantly larger under salt stress.

### 3.4 Differential methylation sequence analysis under salt and alkali stresses

To characterize the methylation -variant sequences in the *T*. *chinensis* genome under stress, 120 and 105 differential fragments were initially recovered. Ultimately, 81 and 68 distinct methylation -variant bands were successfully obtained from salt and alkali stresses treatments, respectively, for subsequent sequencing and alignment analysis. As summarized in **Table 5**, among the sequences identified under salt stress, 12 exhibited high homology to known gene sequences, 9 of which underwent demethylation. Functional annotations revealed that these sequences are involved in a broad spectrum of physiological processes. Specifically, they encompass genes encoding purple acid phosphatases (PAPs), zinc-finger proteins, amino acid transporters, threonine protein kinases, ATP-dependent proteins, and ribonucleoside diphosphate-related enzymes.

**Table 5:** Homology analysis of DNA methylation-variant sequences in *T. chinensis* under salt stress.

Fragment	Methylation Status under Stress	Accession	Description	E-Value	Size (bp)
MS7	Demethylated	KMZ64103.1	Hypothetical protein	2e-06	69
MS22	Demethylated	XP_021612322.2	Amino acid transporter AVT1	7e-17	419
MS29	Demethylated	QSH89921.1	ATP-dependent proteolytic subunit	2e-05	121
MS44	Demethylated	KAD7479233.1	Hypothetical protein	5e-06	72
MS49	Methylated	KAG6603932.1	Purple acid phosphatase 6	8.8e-92	217
MS64	Demethylated	XM_010258498.2	Nelumbo nucifera UDP-galactose	3.6e-21	86
MS87	Demethylated	XR_001278061.2	Probable receptor-like protein	1.2e-36	135
MS92	Demethylated	HQ664664.1	Tamarix pentandra rpl23	1.7e-36	180
MS102	Demethylated	OMO61932.1	Zinc finger,CCCH-type	2e-07	101
MS108	Demethylated	PWA69971.1	Amino acid transporter protein	2e-16	177
MS115	Methylated	LR792835.1	Threonine-protein kinase	1.5e-90	291
MS116	Methylated	MCH98784.1	Ribonucleoside-diphosphate reductase largesubunit-like protein	5e-11	115

In contrast, only 5 differential sequences from the alkali stress group shared high homology with known functional sequences, with 3 being demethylated (**Table 6**). Functional annotations associated these sequences with calcium-dependent protein kinases (CDPKs), membrane metalloproteases, ATP-dependent proteins, and other protein kinases, as well as proteins of unknown function. These sequences may provide preliminary clues for understanding the alkali stress response in *T*. *chinensis*.

**Table 6:** Homology analysis of DNA methylation-variant sequences in T. chinensis under alkali stress.

Fragment	Methylation Status under Stress	Accession	Description	E-Value	Size (bp)
MA28	Methylated	AAQ54491.1	Protein kinase-like	8e-10	101
MA61	Demethylated	KAH9793230.1	Membrane Metalloprotease Arasp	6e-52	333
MA77	Methylated	XP_068481536.1	Uncharacterized protein	6e-06	68
MA91	Demethylated	QSH89921.1	ATP-dependent proteolytic subunit	2e-05	121
MA94	Demethylated	XP_042520892.1	Calcium-dependent protein kinase 13-like	2e-04	131

## 4 Discussion

### 4.1 Salt and alkali stresses induce severe phenotypic damage in *T*. *chinensis* leaves

As a typical “pioneer” tree species with robust resilience, *T*. *chinensis* holds significant value for ecological restoration of saline-alkaline lands, mining stress-resistance genes, and medicinal plant development (Gao et al., 2008; Wang et al., 2014). However, epigenetic mechanisms governing its stress resistance, particularly dynamic genomic DNA methylation patterns under salt and alkali stresses, have remained largely unexplored. In the present study, *T*. *chinensis* exhibited no significant phenotypic deviations from control groups after 5 days of exposure to various salt and alkali concentrations. This observation suggests that short-term stress levels remained below the basal tolerance threshold of this species. By day 10, however, leaf damage manifested under 150 mM alkali stress, whereas comparable symptoms emerged only under a much higher salt concentration (400 mM) (**Figure 1**), indicating significantly greater salt tolerance. Research suggests *T*. *chinensis* maintains K^+^/Na^+^ ratios by accumulating K^+^ and utilizing specific genes for Na^+^ and K^+^ compartmentalization, thereby mitigating ionic toxicity (Liu et al., 2021). Furthermore, Qu et al. found that salt stress increased the relative abundances of carbon- and sulfur-cycling communities in the *T. chinensis* rhizosphere, with more pronounced carbon-cycling shifts potentially alleviating negative salinity impacts (Qu et al., 2024). Despite the absence of overt phenotypic damage during the initial stress phase (10 days), prolonged 30-day exposure ultimately triggered irreversible physiological degradation, characterized by severe chlorosis, yellowing, and desiccation.

### 4.2 DNA methylation variations reveal differential responses of *T*. *chinensis* to salt and alkali stresses

Plants typically develop necessary response mechanisms through long-term environmental acclimation. DNA methylation, a key epigenetic modification, plays an indispensable regulatory role in abiotic stress responses (Lucibelli et al., 2022; Hussain et al., 2025; Lu et al., 2025; Miryeganeh and Armitage, 2025). Extensive research has confirmed that abiotic stress alters whole-genome cytosine methylation levels and distribution patterns, thereby modulating downstream gene expression and conferring adaptive potential to adverse environments (Song et al., 2016; Huang et al., 2025; Ma et al., 2025; Wu et al., 2025; Xin et al., 2025). For instance, drought stress in *Medicago ruthenica* L. resulted in a 4.41% decrease in genomic DNA methylation; notably, key drought-responsive genes, primarily those involved in abscisic acid (ABA) synthesis and proline accumulation, were activated via demethylation, subsequently enhancing drought resistance (Zi et al., 2024). Furthermore, in the salt-tolerant species *Laguncularia racemosa* L., the magnitude of epigenetic plasticity under salt stress significantly exceeded genetic variation, with whole-genome methylation levels decreasing by approximately 15%. This indicates that demethylation is a critical epigenetic regulatory strategy for this species in its response to salt stress (Lira-Medeiros et al., 2010).

MSAP enables detection of methylation status changes at CCGG sites across the genome, and it cannot distinguish the genomic contexts of methylation nor provide single-base resolution. Despite these limitations, MSAP remains a practical and widely used technique for initial epigenetic screening in non-model species, as it does not require a reference genome and allows efficient large-scale assessment of methylation variation patterns. In the present study, MSAP technology was utilized to systematically evaluate the genomic DNA methylation landscapes of *T. chinensis* under varying salt and alkali stresses conditions. By establishing intrinsic correlations between stress factors and methylation variations, our findings provide preliminary evidence for the epigenetic correlates of salt-alkali tolerance in this species. The MSAP data revealed distinct temporal and stress-specific methylation patterns (**Figure 3**), with salt stress favoring progressive demethylation and alkali stress inducing more stable hypermethylation responses. These divergent patterns suggest that *T*. *chinensis* exhibits distinct epigenetic response patterns to adapt to the unique damage characteristics imposed by different stressors.

Integrating our earlier phenotypic observations (**Figure 1**), *T*. *chinensis* exhibited significantly higher salt tolerance than alkali tolerance at equivalent concentrations. This phenotypic divergence appears to be closely linked to the respective methylation patterns. MSAP detection revealed that genomic DNA remodeling was more extensive and active under salt stress. This widespread, demethylation-dominated pattern is potentially associated with salt tolerance, possibly by relieving transcriptional repression and upregulating stress-responsive genes, thereby increasing the capacity for ROS scavenging and the mitigation of ionic toxicity. Furthermore, site-specific analysis indicated that both hypermethylation and demethylation under salt stress occurred primarily at CNG sites, a pattern mirrored by demethylation under alkali stress. This identifies CNG sites as the most sensitive methylation modification loci within the *T*. *chinensis* genome under abiotic stress, consistent with findings in other plant species where CNG motifs are highly susceptible to stress-induced regulation (Yang et al., 2022). Collectively, these findings demonstrate that varying intensities of salt and alkali stresses trigger extensive genomic DNA methylation changes in *T*. *chinensis*, generally manifesting synergistic time-and dosage-dependent effects. While salt stress was characterized by a predominance of DNA demethylation, alkali stress primarily elicited a hypermethylation response. Notably, the magnitude of methylation variation under salt stress was substantially larger than that under alkali stress. These contrasting patterns suggest that *T*. *chinensis* employs distinct DNA methylation responses to different abiotic stresses, particularly in response to salt stress.

### 4.3 Potential functional sequences responding to salt and alkali stresses in *T*. *chinensis*

Functional annotation of differentially methylated sequences provides useful insights into the molecular functions that may be associated with the stress responses of *T*. *chinensis* (**Tables 5**, **6**). The differentially methylated sequences identified in this study were annotated to functional categories including PAPs, zinc-finger proteins, transport proteins, threonine protein kinases, ATP-dependent proteins, and ribonucleoside diphosphate reductases, all of which have been implicated in plant stress responses. Evidence suggests that members of the PAP and zinc -finger protein families are indispensable for plant environmental resilience, primarily by orchestrating ROS scavenging and promoting the biosynthesis of protective metabolites to mitigate oxidative damage (Kang et al., 2019; Yin et al., 2019). Furthermore, amino acid transport proteins, threonine protein kinases, ATP-dependent proteins, and ribonucleoside diphosphates are well-documented regulatory factors in abiotic stress signaling, contributing substantially to growth, development, and long-term environmental adaptation (Hirner et al., 2006; Quan et al., 2007). Consistently, more methylation-variant sequences with known functional annotations were recovered under salt stress (12 sequences, 9 demethylated) than under alkali stress (5 sequences, 3 demethylated), including genes encoding PAPs, zinc-finger proteins, and protein kinases that have been implicated in plant stress responses (Hirner et al., 2006; Quan et al., 2007; Kang et al., 2019; Yin et al., 2019). This is consistent with the more extensive demethylation observed under salt stress and may partially explain the higher salt tolerance of *T*. *chinensis*. Nevertheless, it should be noted that the functional annotations of these differentially methylated fragments are based solely on sequence similarity, and their actual roles in salt or alkali stress tolerance require further experimental validation.

Integrating these methylation-pattern analyses suggests that these key functional sequences likely mediate the adaptation of *T. chinensis* to salinity and alkalinity through dynamic epigenetic modifications. This aligns with findings in plants such as *M*. *ruthenica* and *L*. *racemosa*, where methylation-mediated regulation of stress-resistance genes is a core survival strategy (Lira-Medeiros et al., 2010; Zi et al., 2024). Notably, common epivariant genes (MS29, MA91) were identified in response to both salt and alkali stresses. This intersection suggests that the epigenetic regulatory frameworks for these two stressors are not entirely autonomous but share conserved overlapping mechanisms. As the genomic information for *T*. *chinensis* becomes more complete, further validation of the methylation changes and/or their relationships with gene expression will be possible. In conclusion, these results provide significant epigenetic perspectives on the molecular architecture underlying the robust salt and alkali tolerance of *T*. *chinensis*.

## 5 Conclusions

This study systematically investigated the impacts of varying concentrations and durations of salt and alkali stresses on the phenotypes and genome-wide DNA methylation patterns of *T*. *chinensis*. Our findings reveal that, at equivalent concentrations, *T*. *chinensis* exhibits significantly higher tolerance to salinity than to alkalinity. MSAP analysis characterized divergent genome-wide DNA methylation trends: salt stress was associated with predominantly demethylation events, whereas alkali stress corresponded to hypermethylation, with both types of variation occurring primarily at CNG sites. Furthermore, the extent of DNA methylation variation under salt stress was found to be more extensive and dynamic compared to that under alkali stresses. The recovery and sequencing of methylation -variant bands yielded 81 (salt stress) and 68 (alkali stress) distinct sequences. Among these, 17 sequences were homologous to known stress-responsive genes, including those encoding PAPs, zinc -finger proteins, amino acid transporters, protein kinases, and ATP-dependent proteins. In summary, this study describes phenotypic response characteristics and MSAP-detected DNA methylation variation patterns in *T*. *chinensis* under salt and alkali stresses. These results provide preliminary epigenetic information for understanding the complex mechanisms of salt-alkali tolerance in *T*. *chinensis* and may serve as a reference for future molecular breeding of stress-resistant varieties and ecological restoration of saline-alkaline lands.

## Data Availability

The datasets presented in this study can be found in online repositories. The names of the repository/repositories and accession number(s) can be found in the article/**Supplementary Material**.
