# Supplementary material for: DNA methylation variations induced by salt and alkali stresses and their differential responses in Tamarix chinensis L
Source: Front Plant Sci. 2026 Aug 5;17:1894569. doi: 10.3389/fpls.2026.1894569 (PMC13505199; doi:10.3389/fpls.2026.1894569)
Supplement: Supplementary file 1 [file Table1.docx]

**Supplementary Materials:**

**Table S1.** Sequences of adaptors and primers for MSAP analysis.

| **Adapters(Primers)** | **Sequences（5'-3'）** |
| --- | --- |
| *EcoR*Ⅰ-adapter Ⅰ | CTCGTAGACTGCGTACC |
| *EcoR*Ⅰ-adapter Ⅱ | AATTGGTACGCAGTC |
| H/M-adapter Ⅰ | GATCATGAGTCCTGCT |
| H/M-adapter Ⅱ | CGAGCAGGACTCATGA |
| *EcoR*Ⅰ + A | GACTGCGTACCAATTCA |
| *H/M*+ 0 | ATCATGAGTCCTGCTCGG |
| *EcoR*Ⅰ -AAC | GACTGCGTACCAATTCAAC |
| *EcoR*Ⅰ -AGA | GACTGCGTACCAATTCAGA |
| *EcoR*Ⅰ -AAG | GACTGCGTACCAATTCAAG |
| *EcoR*Ⅰ -ATC | GACTGCGTACCAATTCATC |
| *EcoR*Ⅰ -ACA | GACTGCGTACCAATTCACA |
| *EcoR*Ⅰ -AGG | GACTGCGTACCAATTCAGG |
| *EcoR*Ⅰ -ACT | GACTGCGTACCAATTCACT |
| *EcoR*Ⅰ -AGC | GACTGCGTACCAATTCAGC |
| *EcoR*Ⅰ -ACC | GACTGCGTACCAATTCACC |
| *EcoR*Ⅰ -ACG | GACTGCGTACCAATTCACG |
| *H/M*-TTA | ATCATGAGTCCTGCTCGGTTA |
| *H/M*-TTG | ATCATGAGTCCTGCTCGGTTG |
| *H/M*-TGA | ATCATGAGTCCTGCTCGGTGA |
| *H/M*-TTC | ATCATGAGTCCTGCTCGGTTC |
| *H/M*-TGT | ATCATGAGTCCTGCTCGGTGT |
| *H/M*-TCC | ATCATGAGTCCTGCTCGGTCC |
| *H/M*-TGC | ATCATGAGTCCTGCTCGGTGC |
| *H/M*-TCT | ATCATGAGTCCTGCTCGGTCT |
| *H/M*-TAC | ATCATGAGTCCTGCTCGGTAC |
| *H/M*-TCG | ATCATGAGTCCTGCTCGGTCG |
